# A Genomic Instability Score in Discriminating Nonequivalent Outcomes of BRCA1/2 Mutations and in Predicting Outcomes of Ovarian Cancer Treated with Platinum-Based Chemotherapy

**DOI:** 10.1371/journal.pone.0113169

**Published:** 2014-12-01

**Authors:** Shaojun Zhang, Yuan Yuan, Dapeng Hao

**Affiliations:** 1 College of Bioinformatics Science and Technology, Harbin Medical University, Harbin 150081, P.R. China; 2 Department of Gynecology and Obstetrics, The Forth Affiliated Hospital of Harbin Medical University, Department of Gynecology and Obstetrics, Harbin 150081, P.R. China; Meharry Medical College, United States of America

## Abstract

Detecting mutation in *BRCA1/2* is a generally accepted strategy for screening ovarian cancers that have impaired homologous recombination (HR) ability and improved sensitivity to PARP inhibitor. However, a substantial subset of *BRCA*-mutant ovarian cancer patients shows less impaired or unimpaired HR ability, resulting in nonequivalent outcome after ovarian cancer development. We hypothesize that genomic instability provides a lifetime record of DNA repair deficiency and predicts ovarian cancer outcome. Based on the multi-dimensional TCGA ovarian cancer data, we developed a biological rationale-driven genomic instability score integrating somatic mutation and copy number change in a tumor genome. The score successfully divided *BRCA*-mutant ovarian tumors into cases of significantly improved outcome and cases of unimproved outcome. The score was also capable of discriminating HR-deficiency indicated by *BRCA1* epigenetically silencing, *EMSY* amplification and homozygous deletion of core HR genes. We further found that the score was positively correlated with the complete response rate of chemotherapy and the rate of platinum-sensitivity, and predicted improved outcome of ovarian cancer, regardless of *BRCA*-mutation status. The score may have important value in outcome prediction and clinical trial design.

## Introduction

Both *BRCA1* and *BRCA2* are tumor suppressor genes involved in the repair of DNA double-strand breaks (DSBs) via homologous recombination (HR) [1]. Cells with *BRCA1/2* mutation have an impaired ability to repair DSBs via HR, which is conservative, and potentially error-free, resulting in increased genomic instability and the predisposition to ovarian cancer [2]. It has been hypothesized that ovarian cancer patients with *BRCA1/2* mutation have improved survival because of the sensitivity to specific DNA-damaging agents, such as cisplatin and carboplatin [3], [4]. Furthermore, it has been shown that, given the deficiency of HR, the inhibition of base excision repair pathway by PARP inhibitor usually leads to cell death [5]. This raises hopes to develop targeted therapy for HR deficient ovarian cancers.

However, conflicting results were reported regarding the outcome of ovarian cancer with *BRCA1/2* mutation. Some studies found that the survival of ovarian cancer patients with *BRCA1/2* germline mutation was significantly more favorable than wild-type patients [6], [7], [8], [9], whereas other studies have shown conflicting results [10], [11], [12]. For example, by comparing 37 *BRCA1* mutant ovarian cancer patients with wild-type patients, it has been recently shown that survival of *BRCA1* mutation carriers had no significant difference from wild-type cases [12], [13]. Furthermore, it was found that many *BRCA1* mutant ovarian cancer patients were resistant to chemotherapy agents that induce DSBs [12]. The discrepancy in previous studies indicated that not all ovarian cancer cells with BRCA1/2 mutation exhibited HR deficiency. First, some *BRCA1/2* mutations may not compromise gene function; second, most DNA repair genes are recessive, that is, both alleles should be mutated for the complete loss-of-function [14]. There is no compelling evidence showing that the haploinsufficiency or low expression of *BRCA1/2* gene predicts improved outcome for ovarian cancer [15]. Therefore, new strategies should be developed to identify HR deficient samples.

Genomic instability, as an evolving hallmark of cancer, might have the potential to address the problem. It has been hypothesized that genome instability can be attributed to defects in pathways that maintain genomic stability, especially the HR pathway [16]. In hereditary cancers, the genomic instability has been linked to defects in genes involved in the repair of DSBs via HR, such as *BRCA1/2*, *RAD50* and the Fanconi anaemia gene [17], [18]. Two forms of genomic instability that we consider as reflections of HR deficiency are the chromosomal alteration and the mutator phenotype, which can be quantified by the frequency of copy-number change (CNC) and the frequency of somatic mutation, respectively. The chromosomal alteration can be induced by stalled or collapsed DNA replication forks triggered by oncogenes and mutagenic chemicals, which in turn lead to DSBs [19], [20]. Thus, in HR-deficient cells, the chromosomal alteration accumulates. The absence of HR increases the use of alternative DNA repair pathways, which are mostly error-prone, leading to an increase of sequence mutation and chromosomal translocation [21]. Recently, *Kang et al.* found that high expression of most DNA repair genes, rather than low expression, was associated with improved sensitivity to platinum-based chemotherapy, reflecting an attempt to compensate for the potentially defective HR pathway [15].

In this study, we show that a score constructed by the above two forms of genomic instability can be used to reevaluate the consequences of *BRCA1/2* mutations and to refine HR deficient samples from *BRCA* mutation carriers. Furthermore, it has been suggested that a subset of sporadic ovarian cancer, in the absence of *BRCA1/2* mutation, may harbor HR deficiency and stand to benefit from platinum compounds and PARP inhibitor [22]. Thus, the score may also predict outcome of a large number of ovarian cancer patients, regardless of *BRCA1/2* mutation status.

## Material and Methods

### Ovarian cancer patients

We searched the TCGA database of 325 ovarian cancer patients on November 6, 2012, where both CNC and somatic mutation data were available. Clinicopathological characteristics of ovarian cancer patients, including age, tumor stage and grade and surgical debulking status, are listed in Table 1. All patients received a platinum regimen. 59% of patients achieved a complete response (CR) to adjuvant chemotherapy and 67% of patients with a platinum status were platinum sensitive.

**Table 1:** Clinicopathologic characteristics with different BRCA1/2 status.

Characteristic	All Cases	*BRCA* wild-type	*BRCA1* mutation	*BRCA2* mutation	*BRCA1* methylation	*P* value
**No. of cases**	325	250	42	33	34	
**Age, median [range], y**	59[34–87]	60[34–87]	54[40–79]	55[38–76]	55[40–77]	.92
**Tumor stage**						
**II**	15	12	2	1	2	.26
**III**	255	193	32	29	27	
**IV**	55	45	8	2	5	
**Missing, No.**	1	0	0	1	0	
**Tumor grade**						
**2**	25	19	4	2	2	.46
**3**	292	225	37	30	32	
**Missing, No.**	8	6	0	1	0	
**Residual tumor size, cm**						
**0**	60	42	10	8	8	.99
**<1**	158	120	21	17	18	
**1**–**2**	15	12	2	1	0	
**>2**	57	48	5	4	5	
**Missing, No.**	35	28	4	3	3	
**Response to chemotherapy therapy**						
**CR**	193	140	28	25	22	.55
**Non-CR**	132	110	14	8	12	
**Platinum status**						
**Sensitivity**	132	95	18	19	16	.40
**Resistant**	63	53	7	3	8	

For categorical data (Tumor stage and grade, residual tumor size, response to chemotherapy therapy and platinum status), the Fisher exact test was used to calculate P value in *R*; for continuous variable such as age, the Wilcoxon rank sum test was used in *R*. Patients with debulking status “no macroscopic disease” are labeled as 0 cm in residual tumor size. Number (NO.) depicts the corresponding number of patients in each category. Missing values are excluded from the test analyses. BRCA wild-type cases do not include the BRCA1 methylation cases.

### Construction of a genomic instability score

The genomic instability score for each sample was determined by the number of CNC regions (n_1_) and the number of somatic mutations (n_2_) within a cancer genome, according to the formula: Score  =  K×n_1_ + n_2_. In our study, K was set to 0.5, as it most significantly discriminated between long and short median overall survival in TCGA cohort.

In total, 14970 somatic mutations across 325 ovarian cancer patients were used. These mutations were initially captured by whole-exome sequencing performed on tumors and matched normal controls, and then were validated by low-throughput experiments. Only the validated mutations were used (level3 data from TCGA data portal). All the variant types, including point mutations and indels were put together to construct the score. We further divided the mutations into in-frame mutations and frame-shift mutations and found that both in-frame mutation and frame-shift mutation were significantly predictive of outcome (Figure S1), and thus were put together to construct the score. The log2 ratio of segmented copy numbers between tumor and control DNAs was used to estimate the magnitude of CNC. To reduce the potential noises in CNC data, only the long CNCs regions (>3 Mb, log2ratio>0.05 or <−0.05) were used. This cutoff was selected somewhat arbitrarily, but we found that our results were robust against the exact value of cutoff.

### Selection of HR deficient samples

*BRCA1* hypermethylation, *EMSY* amplification and deficiencies (including non-synonymous mutation and homozygous deletion) in *PTEN*, Fanconi Anemia genes, *RAD* genes and DNA repair genes involved in HR (including *ATM*, *ATR* and *CHEK1/2*) were identified to select HR deficient samples of ovarian cancer. K-means consensus clustering was performed on two-dimensional data of DNA methylation and gene expression to separate *BRCA1* epigenetically silenced tumors from non-silenced tumors. Amplification and homozygous deletion were determined by GISTIC copy number analysis.

### Statistical analysis

The different distribution of the score between HR deficient samples and other samples was assessed by Wilcoxon rank sum test. Survival analyses were conducted by Kaplan-Meier method using the log-rank test. Multivariate analyses were performed by Cox proportional hazards regression model. Overall survival was defined as the time interval from initial surgical excision to death or last follow-up time (censored). The Progression-free survival was defined as the time interval from initial surgical excision to progression (including recurrence and death events) or last follow-up time (censored). All the statistical analyses in this study were two-sided. Significance was defined when the p value was less than 0.05.

## Results

### BRCA1/2 mutation and its association with survival in ovarian cancer

**Figure 1:**
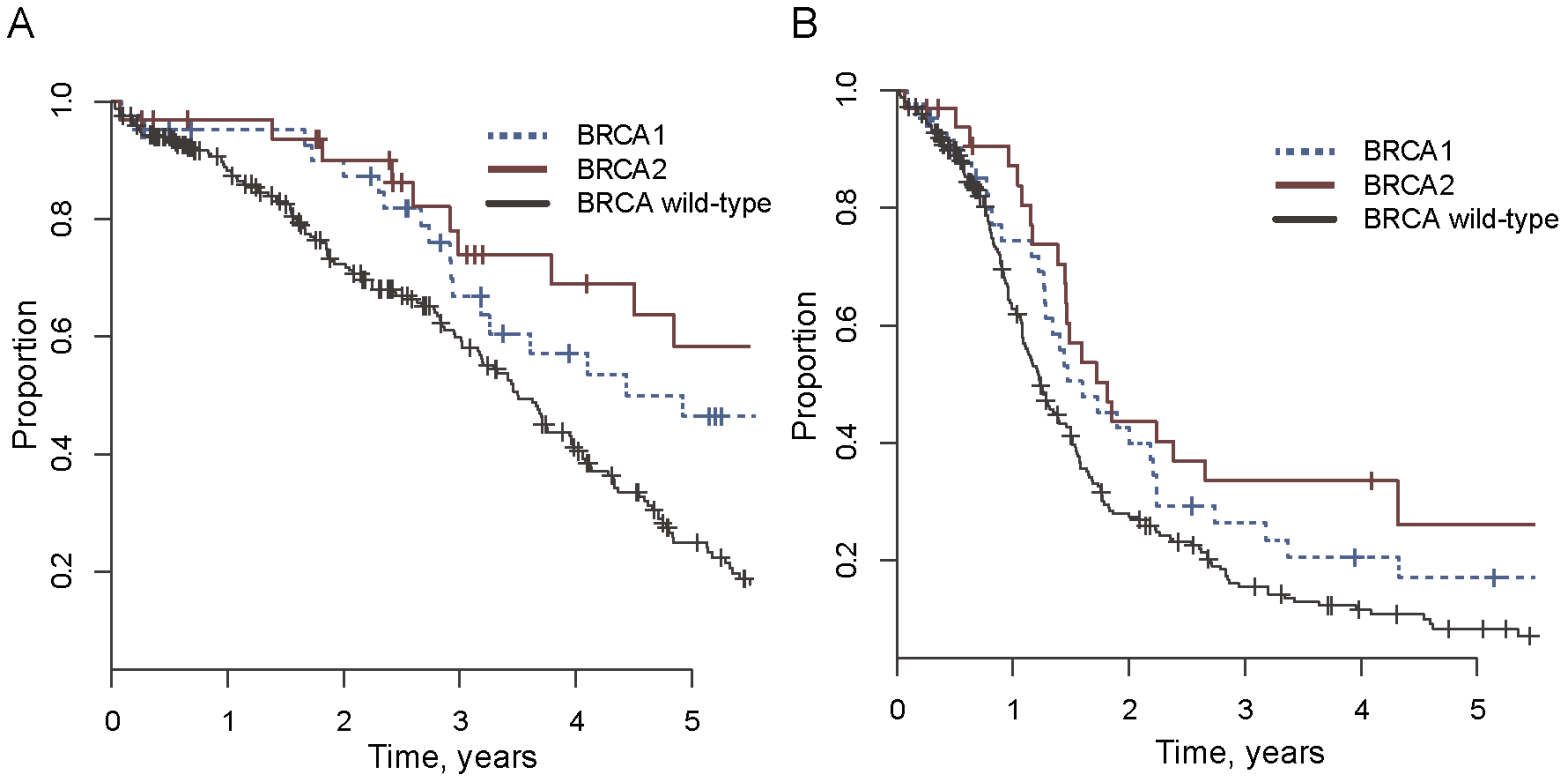
Association of *BRCA1* and *BRCA2* mutations with overall survival (A) and Progression-free survival (B) in ovarian cancer.

According to the updated data in TCGA, *BRCA1* and *BRCA2* were non-synonymously mutated in 42 and 33 ovarian cancer cases, respectively, accounting for 12.9% and 10.1% of 325 patients (Table S1). All but 2 *BRCA1* mutations and 2 *BRCA2* mutations were null mutations (Frame shift or Nonsense). 37 of 42 *BRCA1* mutant ovarian tumors and 29 of 33 *BRCA2* mutant ovarian tumors were used and described in previous studies [12], [13]. Five new *BRCA1* mutant ovarian tumors and four new *BRCA2* mutant ovarian tumors were analyzed compared to the previous studies. Using this updated data, we reevaluated the survival of ovarian patients with *BRCA1/2* mutation and wild-type patients, and revealed different result compared with previous findings [12], [13]. We found that, not only *BRCA2* mutation carriers, but also *BRCA1* mutation carriers had significantly improved survival than wild-type ovarian cancer patients. The 5-year survival rate of *BRCA1* and *BRCA2* mutation carriers was 46% (95% CI, 32%∼68%) and 58% (95% CI, 41%∼83%) respectively, which was significantly higher than 25% (95% CI, 18%∼33%) 5-year survival rate in wild-type patients (Figure 1A; log-rank p = .01 and p = .002, and Cox p = .02 and p = .0007, respectively). The progression-free interval of *BRCA1/2* mutation carriers was also significantly longer than wild-type patients in the multivariate analysis (Cox p = .002 and Cox p = .03 for *BRCA1* mutation and *BRCA2* mutation, respectively; Figure 1B).

### Genomic instability score in predicting outcome of BRCA mutation carriers

**Figure 2:**
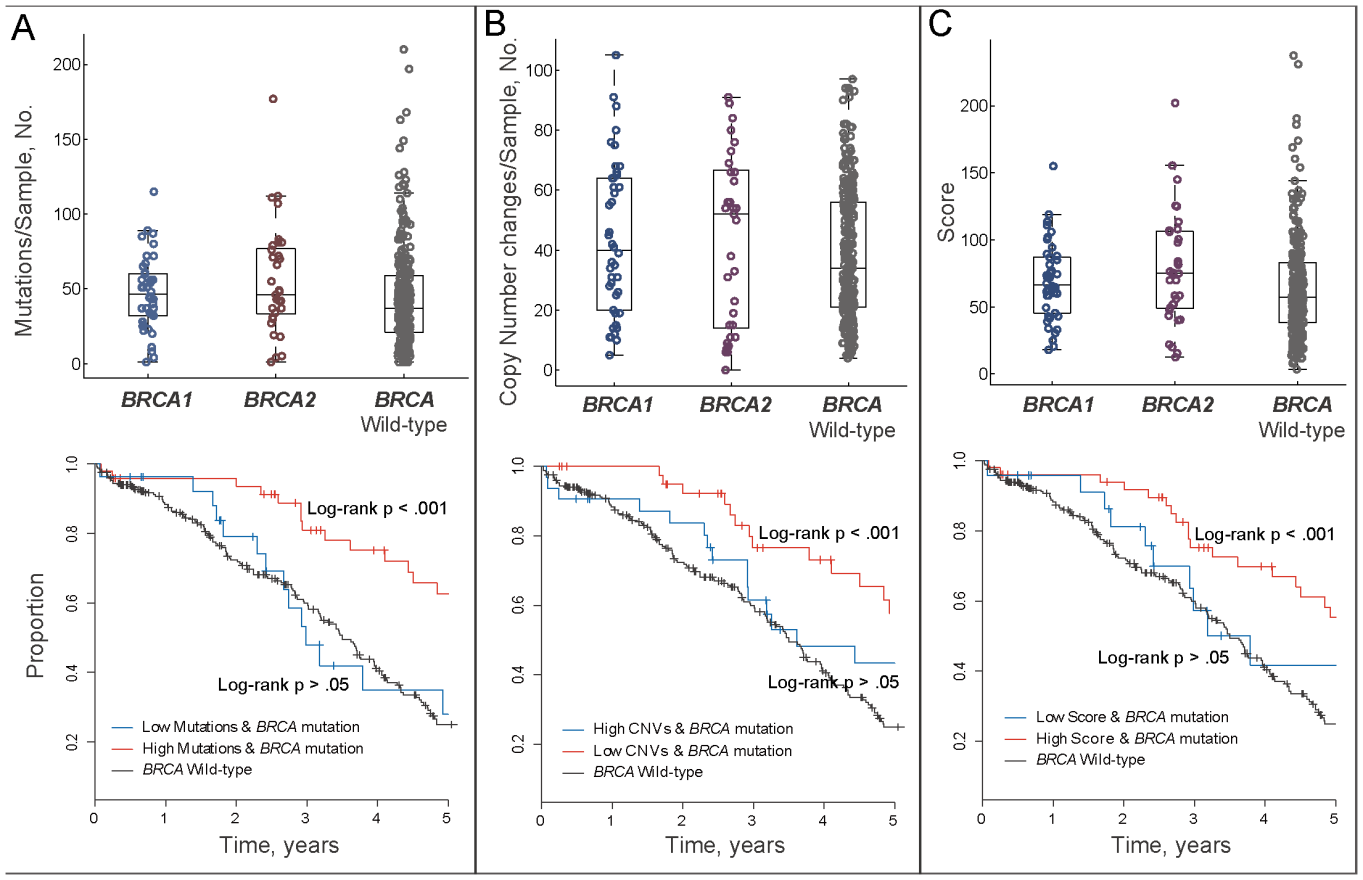
Association of genomic instability with *BRCA1/2* mutations and survival in ovarian cancer. (**A**) Both *BRCA1* and *BRCA2* mutated tumors show elevated level genome mutations. High mutation group of *BRCA1/2* mutated ovarian cancer patients shows significantly improved survival than wild-type patients, whereas low mutation group of *BRCA1/2* mutated patients shows nonsignificant difference compared with wild-type patients. (**B**) Both *BRCA1* and *BRCA2* mutated tumors show increased copy number changes. High CNCs group of *BRCA1/2* mutated ovarian cancer patients shows significantly improved survival than wild-type patients, whereas low CNCs group shows nonsignificant difference compared with wild-type patients. (**C**) Both *BRCA1* and *BRCA2* mutated patients show increased genomic instability score, with *BRCA2* mutated patients show higher score distribution than *BRCA1* mutated patients. High scoring group of *BRCA1/2* mutated patients shows significantly improved survival than wild-type patients, whereas low scoring group shows nonsignificant difference compared with wild-type patients.

To explore the genomic instability of *BRCA* mutated and wild-type ovarian cancer patients, we calculated the frequency of somatic mutation and the frequency of CNC for each tumor genome. Tumors with germline and somatic *BRCA* mutations had no significant differences in outcomes and in genomic instability, and thus were pooled together in down-stream analyses. Both *BRCA1* and *BRCA2* mutated genome showed elevated level of mutation and CNC frequency (Figure 2), being consistent with our hypothesis that HR-deficient pathway leads to an increase of mutation and chromosomal instability. We further noticed that *BRCA2* mutated tumors had higher genomic instability than *BRCA1*-disrupted tumors, suggesting that *BRCA2* mutation carriers exhibited a more severe HR deficiency than *BRCA1* mutation carriers. This was consistent with the higher survival rate of *BRCA2* mutation carriers compared with that of *BRCA1* mutation carriers (Figure 1, significance not achieved due to the small number of samples).

We hypothesized that genomic instability reflects HR deficiency. Based on this hypothesis, ovarian cancer patients carrying *BRCA* mutations were divided into two groups by comparing mutation rate and CNC frequency with the respective median level of wild-type patients. *BRCA* mutation carriers in the high level group of both mutation and CNC showed significantly improved overall survival than wild-type patients (log-rank p<.001 in both cases; Figure 2A-B and Table 2 for multivariable models). In contrast, overall survival of *BRCA* mutation carriers in the low level group of both mutation and CNC was not significantly different from wild-type patients (log-rank p>.05 in both cases). Although the low level group of CNC achieved significance in adjusted model (Table 2), the significance is dramatically lower than the high level group of CNC.

**Table 2:** Cox proportional hazard model using relevant pretreatment factors for patients with different BRCA1/2 mutation status.

Variables	Class	Mutation			Copy Number Variation			Score Value		
		HR(95%CI)	5-Year Rate, % (95% CI)	*P* value	HR (95%CI)	5-Year Rate, % (95% CI)	*P* value	HR(95%CI)	5-Year Rate, % (95% CI)	*P* value
**Genomic instability**	**Wild**	1(reference)	25(19–33)		1(reference)	25(19–33)			25(19–33)	
	**High BRCA**	0.37(0.23–0.58)	63(49–81)	<0.001	0.44(0.27–0.71)	58(42–79)	<0.001	0.44(0.29–0.69)	55(42–74)	<0.001
	**Low BRCA**	0.84(0.26–1.30)	28 (13–61)	0.55	0.53(0.32–0.91)	43(28–68)	0.02	0.59(0.31–1.15)	42(23–76)	0.12
**Grade**	**G2**	1(reference)	60(42–86)		1(reference)	60(42–86)			60(42–86)	
	**G3 and G4**	1.66(0.93–2.97)	29(23–37)	0.08	1.62(0.91–2.91)	29(23–37)	0.10	1.62(0.90–2.90)	29(23–37)	0.10
**Debulking**	**0**–**10 mm**	1(reference)	27(21–36)		1(reference)	27(21–36)			27(21–36)	
	**>10 mm**	1.22(0.86–1.73)	25(15–41)	0.26	1.17(0.83–1.65)	25(15–41)	0.36	1.18(0.84–1.67)	25(15–41)	0.34
**Stage**	**II**	1(reference)	49(25–96)		1(reference)	49(25–96)			49(25–96)	
	**III and IV**	1.55(0.67–3.60)	30(25–38)	0.30	1.60(0.69–3.70)	30(25–38)	0.27	1.60(0.69–3.71)	30(25–38)	0.27
**Age**	**>34**	1.01(1.00–1.03)		0.06			0.04			0.04

Abbreviations: High/Low BRCA, BRCA mutation cases in high/low level group of mutations, CNCs or scores; HR, hazard ratio; CI, confidence interval; Debulking, residual tumor size.

Two-sided *P* values were calculated using Cox regression model adjusting for all the variables in the table.

Patients with debulking status “no macroscopic disease” are labeled as 0 cm.

The significant prognostic value of genomic instability inspired us to develop a score integrating mutation and CNC to identify HR deficient ovarian tumors (Materials and Methods). The score of ovarian cancer patients with *BRCA1*/*2* mutation was significantly higher than wild-type patients (p = .02, Wilcoxon rank sum test, Figure 2C). *BRCA* mutated tumors were then divided into the high score group and the low score group by comparing their scores with the median level of scores of wild-type patients. 30 *BRCA1* and 21 *BRCA2* mutation carriers were divided into the high score group, whereas 12 *BRCA1* and 12 *BRCA2* mutation carriers were divided into the low score group. Tumors in the high level group had significantly higher 5-year survival rate (55%; 95% CI, 42%∼74%) than wild-type patients (log-rank p<.001 and p<.001; Figure 2C and Table 2), whereas tumors in the low level group had no significant difference in survival compared with wild-type patients (log-rank p = .28 and Cox p = .12).

### Genomic instability score is correlated with HR deficiency

**Figure 3:**
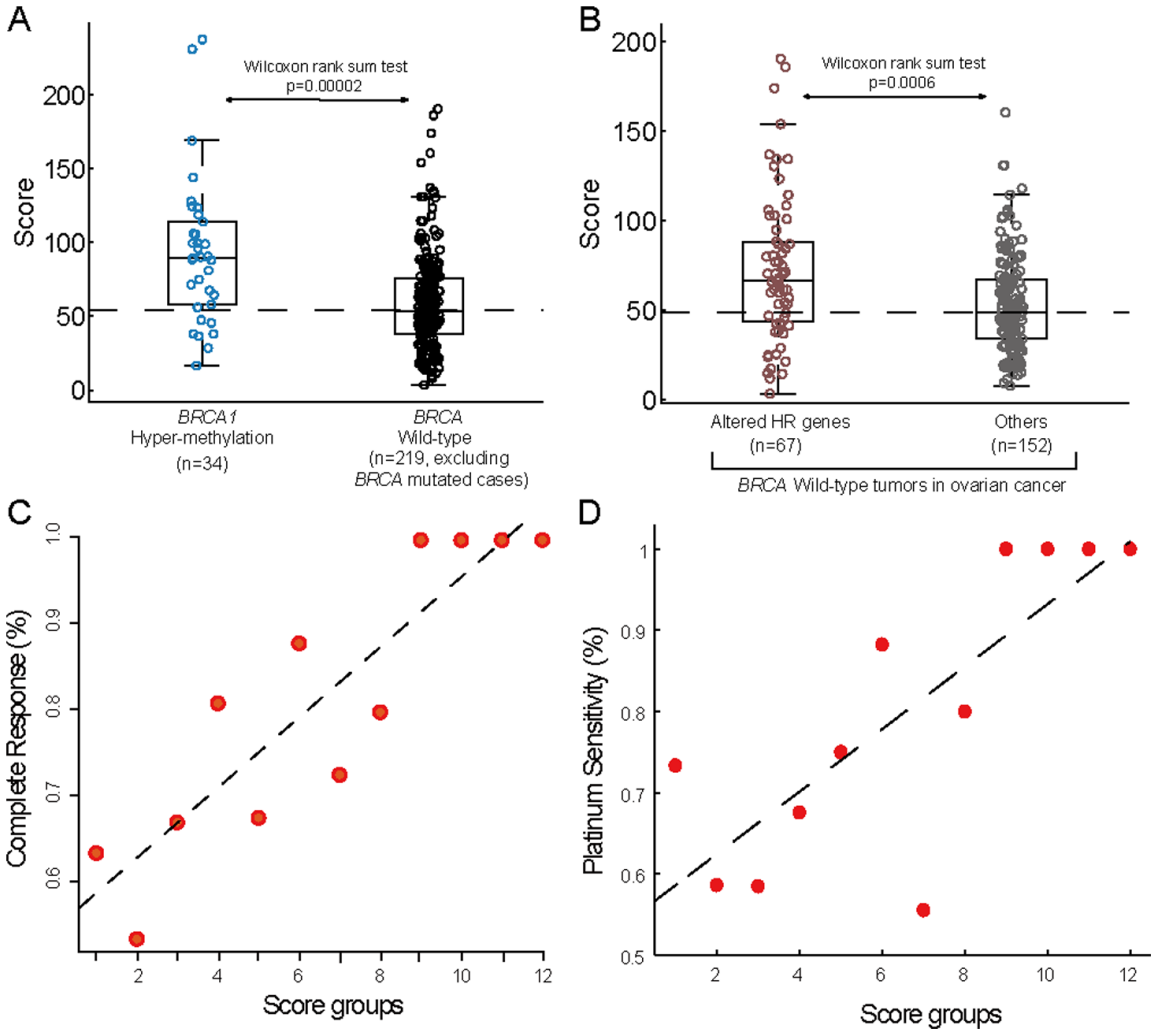
Association of the genomic instability score with HR-deficiency and platinum-response in ovarian cancer. (**A**) The distribution of score in *BRCA1* hyper-methylated patients is significantly higher than that in *BRCA* wild-type patients. (**B**) The distribution of score in HR-deficient patients (including *EMSY* amplification cases, and *PTEN*, Fanconi Anemia genes, *RAD* genes, *ATM*, *ATR* and *CHEK1/2* deficient cases) is significantly higher than that in other *BRCA* wild-type patients. (**C**) Association of the score with complete response (CR). The scores of all patients (n = 325) were divided into 12 equal intervals. The percentage of patients achieving a CR (according to the Response Evaluation Criteria in Solid Tumors) was calculated for patients in each interval and was plotted against each scoring interval in increasing order. Patients in high scoring interval show increasing likelihood of achieving CR. The dashed line represents linear regression line through the data points. (**D**) Same as (**C**) but calculating for platinum-status. 133 platinum-sensitive patients and 62 platinum-resistant patients were analyzed.

Successful separation of *BRCA* mutated tumors by the score may suggest that a substantial subset of *BRCA* mutation carriers show less impaired or unimpaired repair ability via HR. Therefore, it is important to confirm the correlation between our score and other HR related defects in addition to *BRCA1/2* mutation. *BRCA1* epigenetic inactivation has been reported in ovarian cancer [23], [24], and has been recently proven to be a predictor of enhanced sensitivity to platinum-based chemotherapy [25]. We identified 34 *BRCA1* hypermethylated ovarian tumors characterized by both promoter hypermethylation and reduced expression of *BRCA1* (Materials and Methods). A direct comparison between *BRCA1* silenced tumors and BRCA wild-type tumors (Both BRCA mutation samples and *BRCA1* silenced samples were excluded) revealed significant difference in the distribution of the score: average score of *BRCA1* hypermethylated tumors and wild type tumors was 93 and 59, respectively (p = .00002, Wilcoxon rank sum test, Figure 3A).

In addition to *BRCA1/2* deficiency, the amplification of *EMSY* [26] and deficiencies in *PTEN* [27], Fanconi Anemia genes [3], *RAD* genes and DNA repair genes involved in HR (including *ATM*, *ATR* and *CHEK1/2*) have also been identified to cause HR defects in human cancer [28]. To explore whether the score could discriminate HR deficient samples from *BRCA* wild-type ovarian cancer patients, we examined the *BRCA* wild-type tumors and identified 67 tumors for which at least one of those aforementioned genes was altered and 152 tumors for which none of the genes were altered (Materials and Methods and Table S2). Average score of the 67 HR deficient samples and the other 152 samples was 73 and 54, respectively (p = .0006, Wilcoxon rank sum test, Figure 3B).

### Probability of achieving CR and platinum status based on genomic instability score

Overall, 59.4% of patients (193 of 325) in TCGA ovarian cancer cohort achieved a CR to adjuvant chemotherapy (Table 1). To explore whether genomic instability score correlates with the probability of CR, we divided the score into 12 equal intervals and plotted the percentage of patients achieving a CR against each interval of increasing scores. A strong correlation was observed between the score and the likelihood of achieving CR (Figure 3C).

We further investigated whether the score could correlate with platinum status of ovarian tumors. Overall, 133 ovarian cancer patients were platinum sensitive and 62 patients were platinum resistant (Table 1). As shown in Figure 3D, a strong correlation was observed between the increasing score and likelihood of platinum sensitivity. We found that only 25.5% of patients with score higher than the median score of patients with known platinum status were platinum resistant, whereas 39.2% of patients with score lower than the median score were platinum resistant (Fisher exact test, p = 0.05).

### Relationship between genomic instability score and clinical outcome in ovarian cancer patients

These data suggested that the score might be predictive of survival for a large number of ovarian cancer patients, regardless of *BRCA1/2* mutations. To test this, we divided all the 325 ovarian tumors into two groups by the median score—tumors with low scores (<60) and tumors with high scores (> = 60). This natural cut point was used because it divided patients into two groups with an equal number of samples, and seemed to be the simplest way for clinical application. No significant difference in clinical characteristics including age, stage, grade and residual tumor size between the high score group and the low score group was observed (Table 3). The percentage of patients that were disease-free in five years in high-score group and low-score group was 17% and 7%, respectively (p<.05, Fisher exact test).

**Table 3:** Association of high vs low scoring subgroup with clinical characteristics.

Characteristic	High Scoring Group (n = 168)		Low Scoring Group (n = 157)		P value
	No.	%	No.	%	
**Age, mean [range]**	58.5 [39–87]		60 [34–87]		.78
**Tumor stage**					
**II**	11	6.5	4	2.55	.21
**III**	128	76.2	126	80.3	
**IV**	29	17.3	26	16.6	
**Missing, No.**	0	0	1	0.6	
**Tumor grade**					
**2**	12	7.3	13	8.3	.06
**3**	153	92.7	139	88.5	
**Missing, No.**	0	0	5	3.2	
**Residual tumor size, cm**					
**0**	37	22	23	14.6	.48
**<1**	76	45.2	82	52.2	
**1**–**2**	7	4.2	8	5.1	
**>2**	29	17.3	28	17.8	
**Missing, No.**	19	11.3	16	10.2	

**Figure 4:**
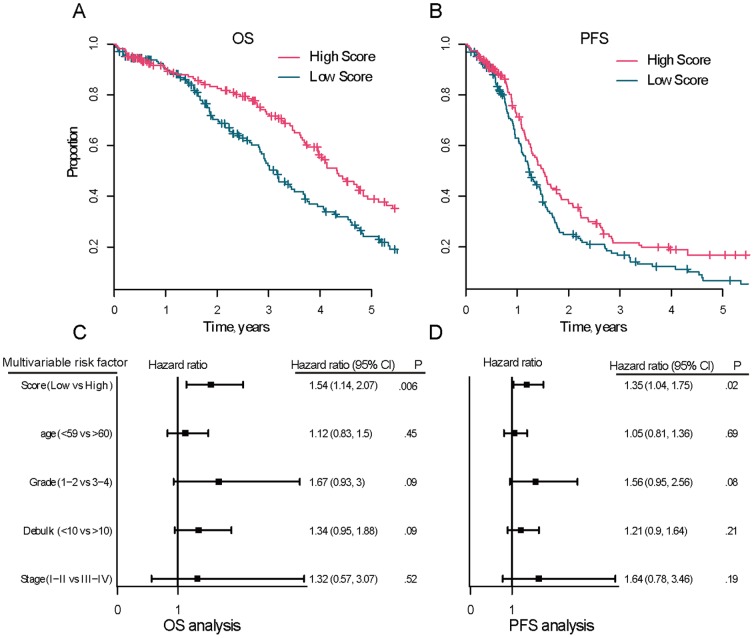
Ability of the genomic instability score to predict overall survival (OS) and progression-free survival (PFS) of ovarian cancer. The TCGA dataset with somatic mutations and CNCs (n  =  325) were analyzed and were divided into low and high scoring groups. Kaplan-Meier analysis was used to assess the OS (**A**) and PFS (**B**) in low versus high scoring group (log-rank p  = .004 and p  = .009, respectively). Multivariable analysis was performed using the Cox proportional hazards model to ensure that the score was independently prognostic for OS (**C**) and PFS (**D**). Solid squares represent the hazard ratio and the horizontal lines represent 95% confidence intervals (CI) of hazard ratios.

The score was capable of discriminating between long and short median overall survival: the ovarian cancer patients in the high-score group and the low-score group had median overall survival of 4.3 years and 3.2 years, respectively (log-rank p = .004, Figure 4A). The 5-year survival rate for the high-score group and the low-score group was 38% and 25% (p  = .07, Fisher exact test), respectively. Finally, samples in the high-score group had significantly longer progression-free survival (PFS) than samples in the low-score group (5-year PFS rate of high-score vs. low-score: 17% vs. 7%, log-rank p  = .009; Figure 4B).

In univariate analysis, high score predicted both improved overall survival and PFS while low score predicted both worse overall survival and PFS (low versus high scores, HR  =  1.52, 95% CI  =  1.14 to 2.03, p  = .005 for overall survival and HR  =  1.40, 95% CI  =  1.08 to 1.8**, p  = .01 for PFS). In multivariate analysis adjusting for age, grade, stage and residual tumor size, the hazard ratios of low versus high scores for overall survival and PFS were 1.52 (p  = .006; 95% CI  =  1.13 to 2.05; Figure 4C) and 1.35 (p  =  0.02; 95% CI  =  1.05to 1.75; Figure 4D), respectively, demonstrating that the score maintained an independent association with overall survival and PFS. We also compared the outcome predictive power of our score with CNC and somatic mutation rate respectively, and found our score outperformed the method using only CNC data or mutation data (HR  =  1.32, p  =  0.04 and HR  =  1.38, p  =  0.07, respectively; Figure S2).

## Discussion

DSBs are the most cytotoxic forms of DNA damage [21]. Impaired ability to repair DSBs leads to increased mutations and gross chromosomal alterations, and in turn can be used as targets for cancer therapy [29]. Two main DNA repair pathways have been found so far to repair DSBs: HR and nonhomologous end-joining (NHEJ) [30]. NHEJ is the major pathway to repair DSBs in the absence of HR and is prone to generate mutations at the joining sites [30], [31], [32]. Moreover, because there is no homologous sequence being used as a template to ensure that the two ends being joined are come from contiguous sequence, NHEJ may prone to yield chromosomal deletions and insertions as well [21]. The trick of HR-based repair is using undamaged homologous sequence in sister chromatid to avoid such errors, which is highly reliant on the intactness of *BRCA1/2* proteins [5]. Therefore, in the absence of HR, the mutation and CNC ensue, which can be used as signatures of HR deficiency to benefit clinical outcome prediction.

Recent studies have used the genome instability to predict the outcome and to define HR deficient samples [33], [34], [35], [36], [37]. However, most of these studies were merely based on the copy number data or based on *BRCA1/2* mutations. For example, Baumbusch LO et al. used the total aberration level of copy number to predict the outcome of ovarian cancer [37]. Abkevich V et al. used the correlation between the number of loss of long copy number regions and *BRCA1/2* mutation to predict the outcome of ovarian cancer [34]. Different with these studies, our genome instability score combined copy number variation and genome mutation, which improves the predictive power of clinical outcome compared to using only copy number data (Figure S2). Furthermore, as we have demonstrated, conflicting results were frequently reported regarding the outcomes of *BRCA1/2* mutant patients with ovarian cancer, suggesting that it was not a robust measure to define HR deficiency. Different with the previous studies defining the HR deficiency score based on the BRCA1/2 mutation, our score is based on genome instability. Therefore, our score can be used to further divide *BRCA*-mutant ovarian tumors into cases of significantly improved outcome and cases of unimproved outcome.

The prognostic value of the score is particularly important for ovarian cancer patients who received a standard platinum-based therapy. Many ovarian cancer patients, including *BRCA* mutant patients, are finally identified to be chemotherapy resistant only after having undergone multiply cycles of toxic therapy with little benefit [38]. Therefore, the genomic instability score may have important implication in identifying patients with unfavorable outcome and redirect them to alternate therapies that are more efficacious, such as radiation or other agents (i.e., topotecan) [39], [40].

Detecting *BRCA1/2* mutations is a generally accepted strategy for predicting early breast cancer. Women carrying germline mutations in either of the two genes confer a lifetime risk of 60–85% of developing breast cancer (mostly basal-like) [41], [42]. This indicates that HR deficiency probably underlies the cancer predisposition of breast cancer too. It has been hypothesized that a substantial subset of sporadic breast cancer may harbor HR deficiency. Therefore, the score may also have the potential to identify a larger subset of HR deficient breast cancer patients and redirect them into chemotherapies that may be more efficacious. However, according to TCGA, only a small subset of breast tumors where both mutation data and copy number change data were available, and few of them received a standard platinum-based chemotherapy, which were not enough for a reliable validation.

This study has a few limitations. Although, to our knowledge, the TCGA ovarian cancer cohort represents the largest dataset that is unprecedented in size and in comprehensiveness, we did not find an appropriate independent dataset to validate our results. However, the construction of the genomic instability score was basically independent of the clinical outcome, and was biological hypothesis-driven. Therefore, we believe that this ensured the reproducibility of the score. In addition, detecting high-confidence sequence mutation is still expensive, which may limit its application on clinical prediction. Therefore, we examined the predictive power of invalidated mutation data (level 2) that are generated by whole-exome sequencing, and found that these data also significantly predicted the outcome of ovarian cancer (Figure S3). With further prospective validation on more comprehensive data, the score may have important implication in clinical prediction and in discriminating the function of *BRCA1/2* mutations.

## Supporting Information

Figure S1 Both frame-shift mutations (**A**) and in-frame mutations (**B**) are predictive of outcome of ovarian cancer (log-rank p = .01 and p = .03, respectively).
(PDF)

Figure S2 Ability of the copy number variation and genome mutation to predict outcome of ovarian cancer. (**A**) The patients in the low-CNC group and the high-CNC group had median overall survival of 1167 days and 1511 days, respectively (log-rank p = 0.03). The 5-year survival rates for low-score group and high-score group were 26.1% and 37.6%, respectively. (B) The patients in the low-mutation group and the high-mutation group had median overall survival of 1213 days and 1499 days, respectively (log-rank p = 0.04). The 5-year survival rates for low-score group and high-score group were 26.6% and 36.3%, respectively. Multivariable analysis was performed using the Cox proportional hazards model to ensure that the CNC (**C**) and mutation rate (**D**) were independently prognostic for overall survival. Solid squares represent the hazard ratio and the horizontal lines represent 95% confidence intervals (CI) of hazard ratios.
(PDF)

Figure S3 Ability of unvalidated mutation data in predicting outcome of ovarian cancer: patients in high-mutation group and the low-mutation group had median overall survival of 4.1 years and 3.2 years, respectively (log-rank p = .001). The 5-year survival rates for high-score group and low-score group were 40.3% and 23.7%, respectively.
(PDF)

Table S1 BRCA1/2 mutations of TCGA ovarian cancer patients
(DOC)

Table S2 HR-deficient ovarian cancer samples as indicated by *EMSY* amplification and deficiencies in *PTEN*, Fanconi Anemia genes, *RAD* genes and DNA repair genes involved in HR (including *ATM*, *ATR* and *CHEK1/2*).
(XLS)
