# Supplementary figures and images for: A Genomic Instability Score in Discriminating Nonequivalent Outcomes of BRCA1/2 Mutations and in Predicting Outcomes of Ovarian Cancer Treated with Platinum-Based Chemotherapy
Source: PLoS One. 2014 Dec 1;9(12):e113169. doi: 10.1371/journal.pone.0113169 (PMC4249855; doi:10.1371/journal.pone.0113169)

**A**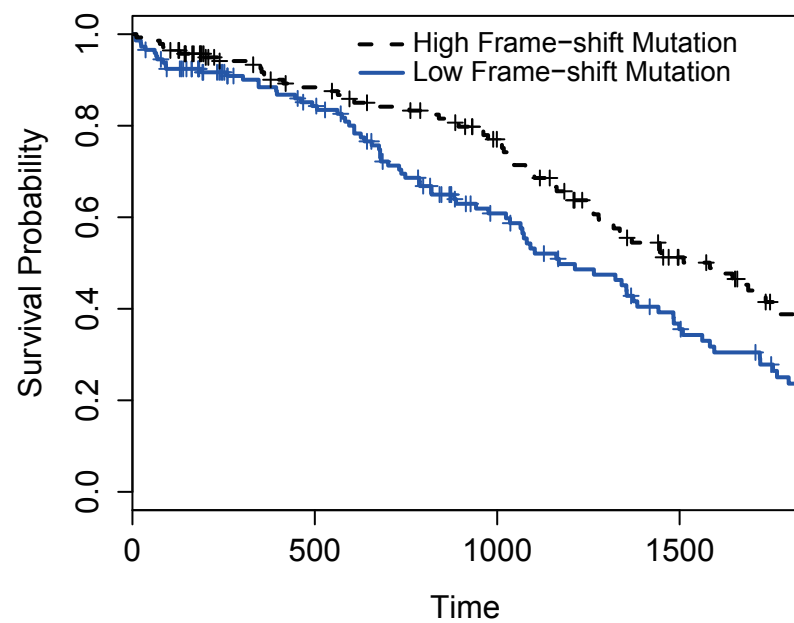**B**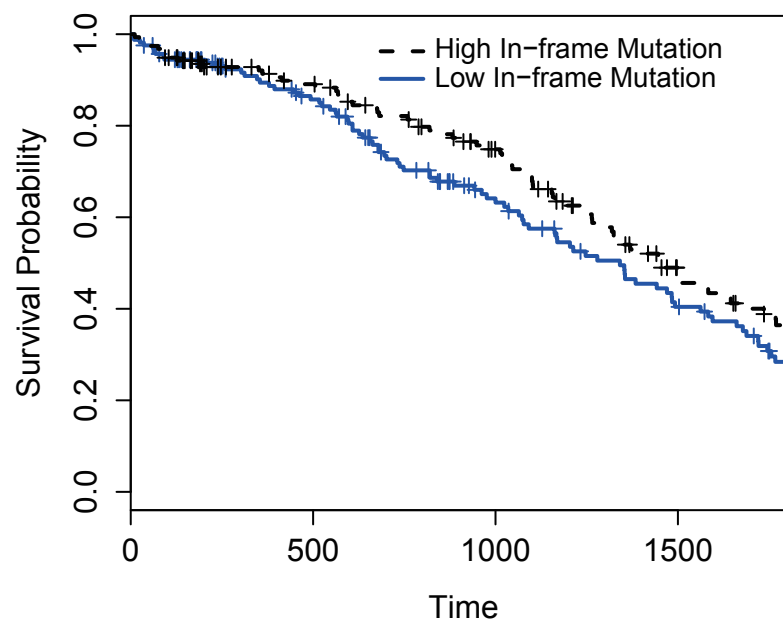

Supplement: Figure S1 — Both frame-shift mutations (A) and in-frame mutations (B) are predictive of outcome of ovarian cancer (log-rank p = .01 and p = .03, respectively). (PDF) [file pone.0113169.s001.pdf]

A

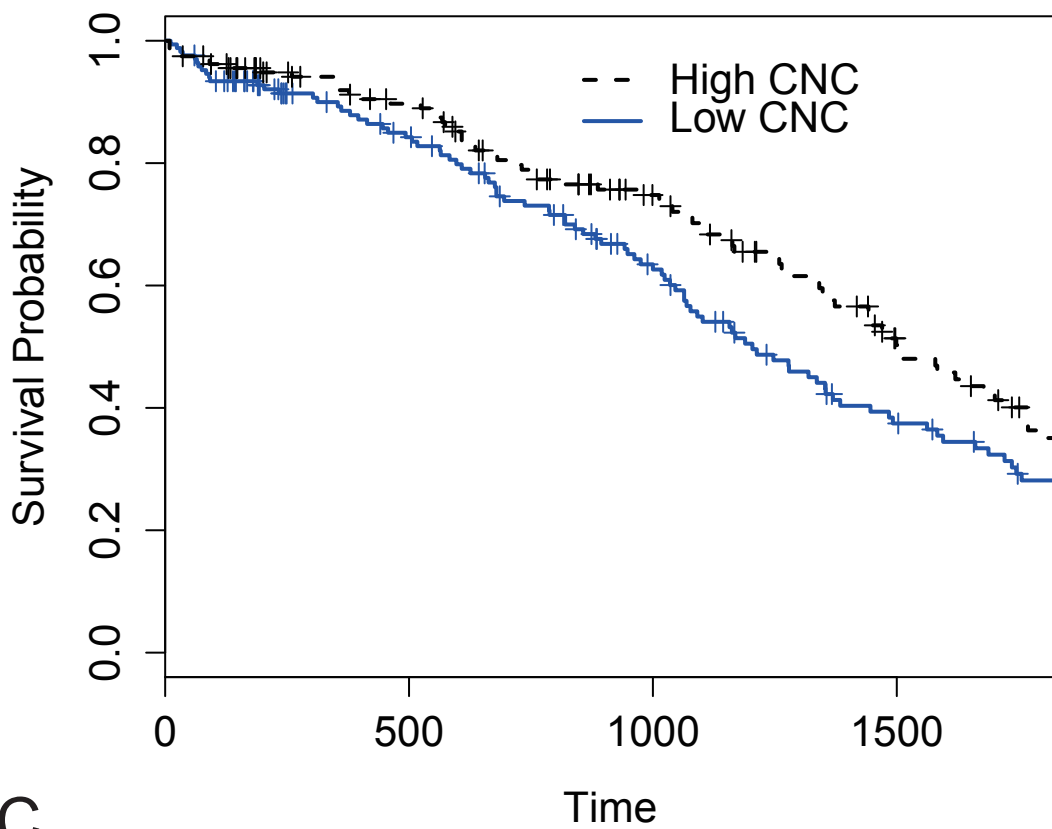

B

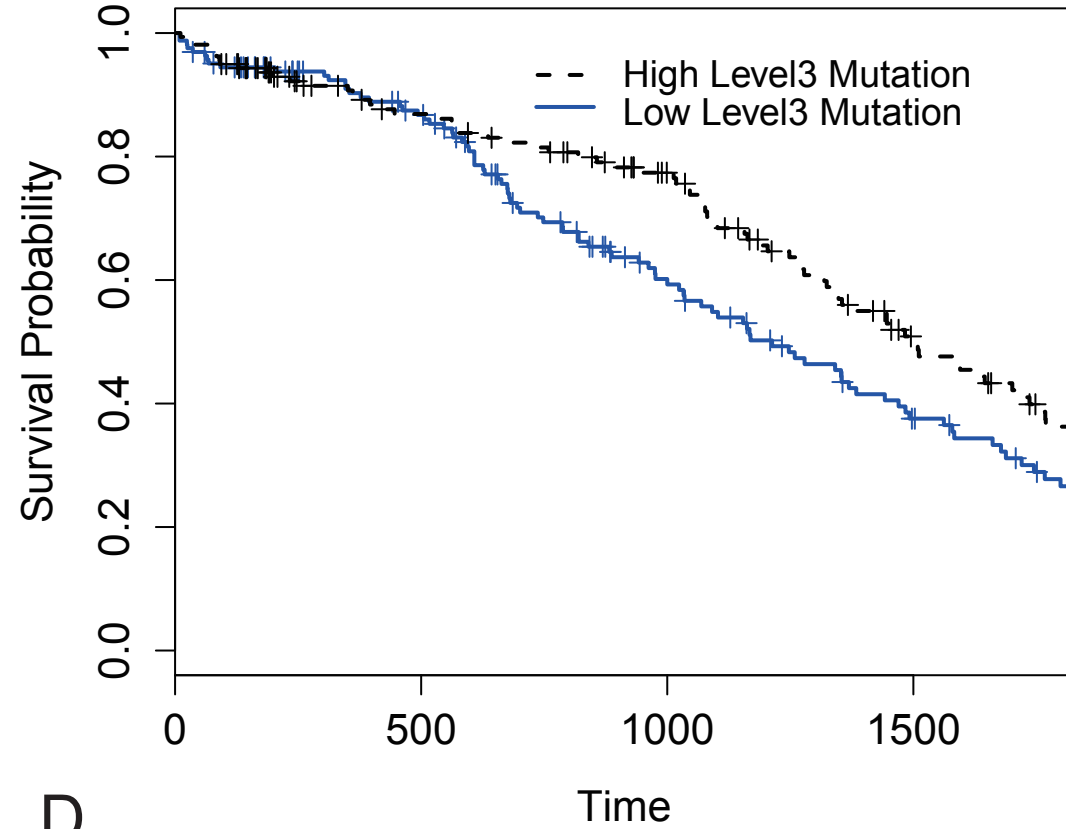

C

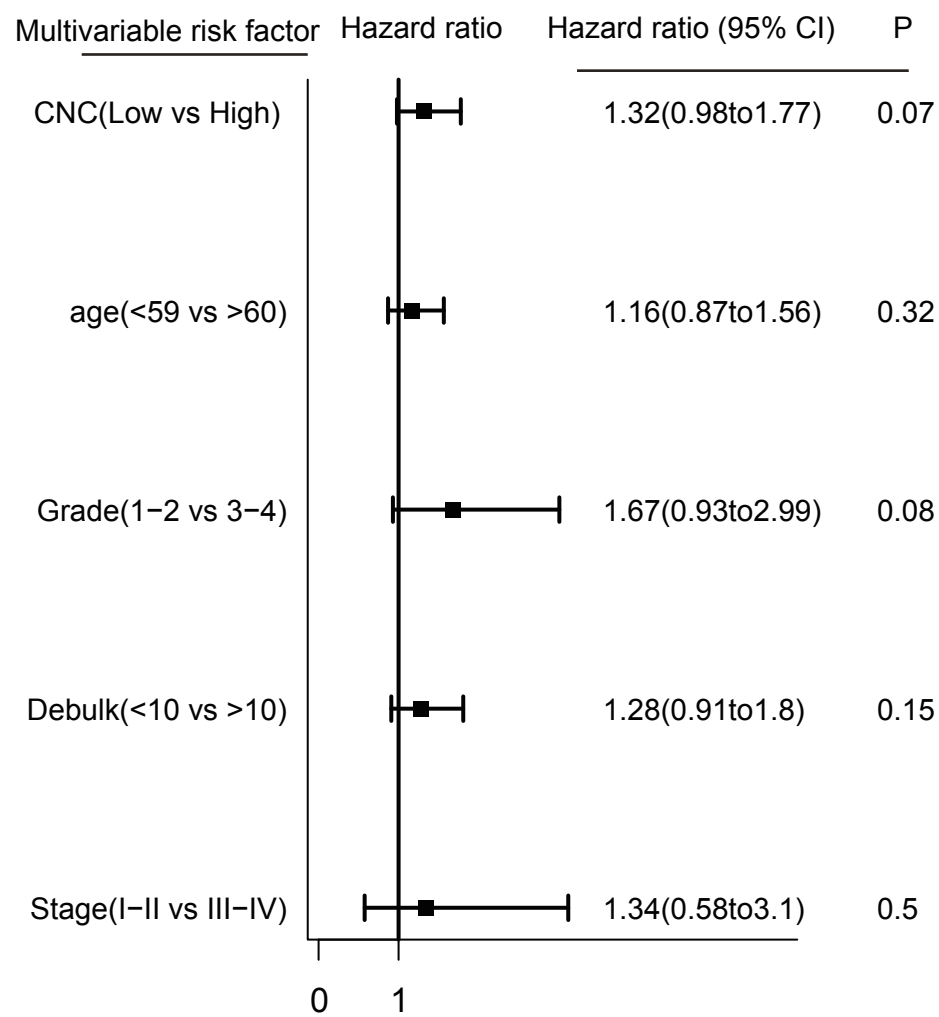

D

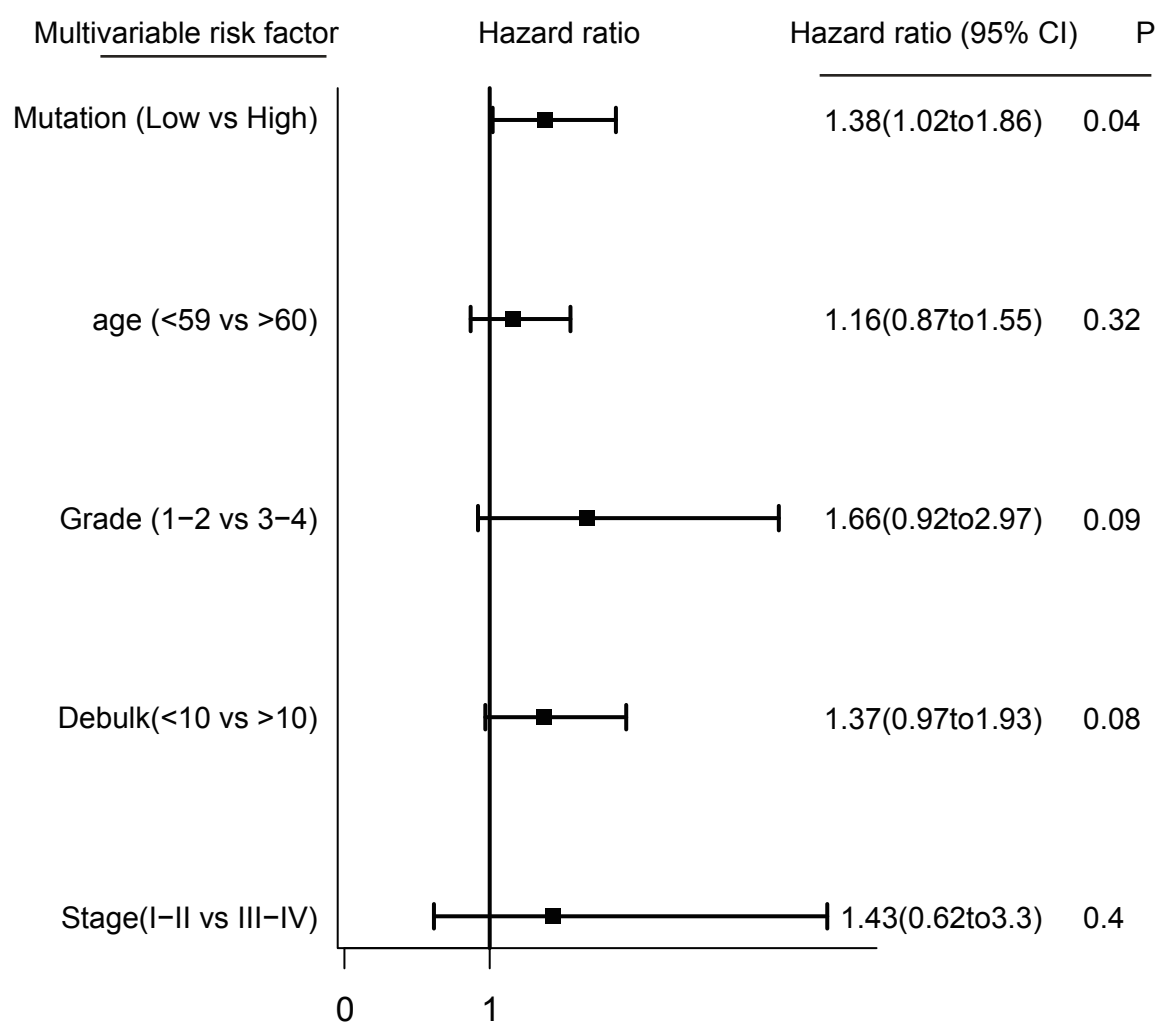

Supplement: Figure S2 — Ability of the copy number variation and genome mutation to predict outcome of ovarian cancer. (A) The patients in the low-CNC group and the high-CNC group had median overall survival of 1167 days and 1511 days, respectively (log-rank p = 0.03). The 5-year survival rates for low-score group and high-score group were 26.1% and 37.6%, respectively. (B) The patients in the low-mutation group and the high-mutation group had median overall survival of 1213 days and 1499 days, respectively (log-rank p = 0.04). The 5-year survival rates for low-score group and high-score group were 26.6% and 36.3%, respectively. Multivariable analysis was performed using the Cox proportional hazards model to ensure that the CNC (C) and mutation rate (D) were independently prognostic for overall survival. Solid squares represent the hazard ratio and the horizontal lines represent 95% confidence intervals (CI) of hazard ratios. (PDF) [file pone.0113169.s002.pdf]

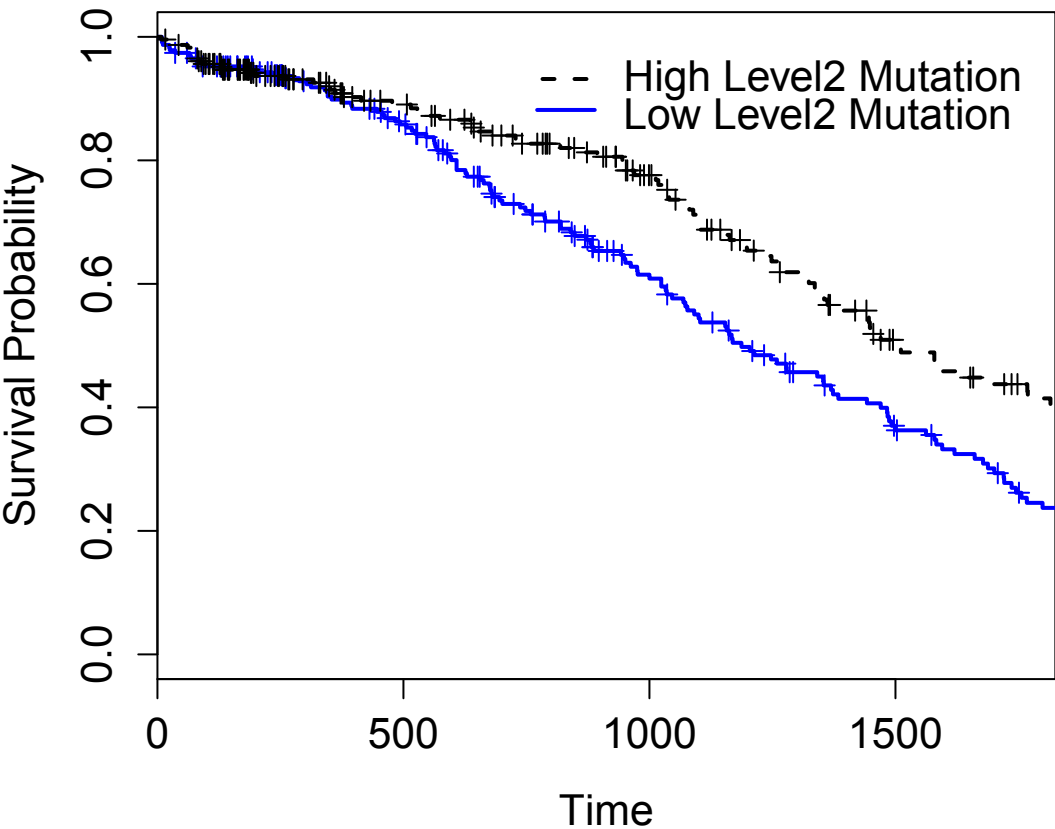

Supplement: Figure S3 — Ability of unvalidated mutation data in predicting outcome of ovarian cancer: patients in high-mutation group and the low-mutation group had median overall survival of 4.1 years and 3.2 years, respectively (log-rank p = .001). The 5-year survival rates for high-score group and low-score group were 40.3% and 23.7%, respectively. (PDF) [file pone.0113169.s003.pdf]
