# Supplementary material for: A Genomic Instability Score in Discriminating Nonequivalent Outcomes of BRCA1/2 Mutations and in Predicting Outcomes of Ovarian Cancer Treated with Platinum-Based Chemotherapy
Source: PLoS One. 2014 Dec 1;9(12):e113169. doi: 10.1371/journal.pone.0113169 (PMC4249855; doi:10.1371/journal.pone.0113169)
Supplement: Table S1 — BRCA1/2 mutations of TCGA ovarian cancer patients (DOC) [file pone.0113169.s004.doc]

**Supplementary Table 1. BRCA1/2 mutations of TCGA patients**

| BRCA Mutations | | | |
| --- | --- | --- | --- |
| BRCA | Cell Type | **Mutation Type** | **Case ID** |
| BRCA1 | Germline | Splice_Site | TCGA-13-1494-01A-01W-0545-08 |
| BRCA1 | Germline | Splice_Site | TCGA-13-2065-01A-01D-1526-09 |
| BRCA1 | Germline | Nonsense_Mutation | TCGA-10-0927-01A-02W-0419-10 |
| BRCA1 | Germline | Nonsense_Mutation | TCGA-10-0937-01A-02W-0419-10 |
| BRCA1 | Germline | Nonsense_Mutation | TCGA-13-0802-01A-01W-0370-10 |
| BRCA1 | Germline | Nonsense_Mutation | TCGA-13-0889-01A-01W-0419-10 |
| BRCA1 | Germline | Nonsense_Mutation | TCGA-13-1408-01A-01W-0490-10 |
| BRCA1 | Germline | Nonsense_Mutation | TCGA-23-1023-01A-03W-0484-10 |
| BRCA1 | Germline | Nonsense_Mutation | TCGA-25-1318-01A-01W-0490-10 |
| BRCA1 | Germline | Nonsense_Mutation | TCGA-59-2348-01A-01W-0799-08 |
| BRCA1 | Germline | Nonsense_Mutation | TCGA-61-2008-01A-02W-0722-08 |
| BRCA1 | Germline | Frame_Shift_Ins | TCGA-09-2051-01A-01W-0799-08 |
| BRCA1 | Germline | Frame_Shift_Ins | TCGA-13-0883-01A-02W-0420-08 |
| BRCA1 | Germline | Frame_Shift_Ins | TCGA-13-0893-01B-01W-0494-09 |
| BRCA1 | Germline | Frame_Shift_Ins | TCGA-23-1122-01A-01W-0486-08 |
| BRCA1 | Germline | Frame_Shift_Ins | TCGA-23-2077-01A-01W-0722-08 |
| BRCA1 | Germline | Frame_Shift_Ins | TCGA-23-2081-01A-01W-0722-08 |
| BRCA1 | Germline | Frame_Shift_Ins | TCGA-24-2298-01A-01W-0799-08 |
| BRCA1 | Germline | Frame_Shift_Ins | TCGA-25-2401-01A-01W-0799-08 |
| BRCA1 | Germline | Frame_Shift_Ins | TCGA-61-1725-01A-01W-0639-09 |
| BRCA1 | Germline | Frame_Shift_Ins | TCGA-61-2610-02A-01W-1092-09 |
| BRCA1 | Germline | Frame_Shift_Del | TCGA-04-1356-01A-01W-0492-08 |
| BRCA1 | Germline | Frame_Shift_Del | TCGA-09-1669-01A-01W-0615-10 |
| BRCA1 | Germline | Frame_Shift_Del | TCGA-09-2045-01A-01W-0799-08 |
| BRCA1 | Germline | Frame_Shift_Del | TCGA-10-0931-01A-01W-0420-08 |
| BRCA1 | Germline | Frame_Shift_Del | TCGA-13-0887-01A-01W-0421-09 |
| BRCA1 | Germline | Frame_Shift_Del | TCGA-13-0903-01A-01W-0421-09 |
| BRCA1 | Germline | Frame_Shift_Del | TCGA-13-1512-01A-01W-0545-08 |
| BRCA1 | Germline | Frame_Shift_Del | TCGA-20-1687-01A-01W-0633-09 |
| BRCA1 | Germline | Frame_Shift_Del | TCGA-23-1027-01A-02W-0486-08 |
| BRCA1 | Germline | Frame_Shift_Del | TCGA-23-1118-01A-01W-0488-09 |
| BRCA1 | Germline | Frame_Shift_Del | TCGA-23-2078-01A-01W-0722-08 |
| BRCA1 | Germline | Frame_Shift_Del | TCGA-23-2079-01A-01W-0722-08 |
| BRCA1 | Germline | Frame_Shift_Del | TCGA-24-1470-01A-01W-0553-09 |
| BRCA1 | Germline | Frame_Shift_Del | TCGA-25-2392-01A-01W-0799-08 |
| BRCA1 | Germline | Frame_Shift_Del | TCGA-29-1688-01A-01W-0633-09 |
| BRCA1 | Germline | Frame_Shift_Del | TCGA-29-1696-01A-01W-0633-09 |
| BRCA1 | Germline | Frame_Shift_Del | TCGA-29-1770-01A-01W-0633-09 |
| BRCA1 | Germline | Frame_Shift_Del | TCGA-29-1775-01A-01W-0639-09 |
| BRCA1 | Germline | Frame_Shift_Del | TCGA-29-1781-01A-01W-0633-09 |
| BRCA1 | Germline | Frame_Shift_Del | TCGA-36-2530-01A-01D-1526-09 |
| BRCA1 | Germline | Frame_Shift_Del | TCGA-42-2582-01A-01D-1526-09 |
| BRCA1 | Germline | Frame_Shift_Del | TCGA-42-2589-01A-01D-1526-09 |
| BRCA1 | Germline | Frame_Shift_Del | TCGA-57-1582-01A-01W-0615-10 |
| BRCA1 | Germline | Frame_Shift_Del | TCGA-61-2109-01A-01W-0722-08 |
| BRCA2 | Germline | Frame_Shift_Del | TCGA-25-1318-01A-01W-0490-10 |
| BRCA2 | Germline | Frame_Shift_Del | TCGA-57-1584-01A-01W-0615-10 |
| BRCA2 | Germline | Frame_Shift_Del | TCGA-25-1318-01A-01W-0490-10 |
| BRCA2 | Germline | Frame_Shift_Del | TCGA-13-0766-01A-02W-0371-08 |
| BRCA2 | Germline | Frame_Shift_Del | TCGA-24-2288-01A-01W-0799-08 |
| BRCA2 | Germline | Frame_Shift_Del | TCGA-25-2404-01A-01W-0799-08 |
| BRCA2 | Germline | Frame_Shift_Del | TCGA-24-2024-01A-02W-0722-08 |
| BRCA2 | Germline | Frame_Shift_Del | TCGA-13-0913-01A-01W-0420-08 |
| BRCA2 | Germline | Frame_Shift_Del | TCGA-13-0886-01A-01W-0420-08 |
| BRCA2 | Germline | Frame_Shift_Del | TCGA-24-2280-01A-01W-0799-08 |
| BRCA2 | Germline | Frame_Shift_Del | TCGA-59-2351-01A-01W-0799-08 |
| BRCA2 | Germline | Frame_Shift_Del | TCGA-29-1764-01A-01W-0633-09 |
| BRCA2 | Germline | Frame_Shift_Del | TCGA-24-1417-01A-01W-0549-09 |
| BRCA2 | Germline | Frame_Shift_Del | TCGA-04-1336-01A-01W-0488-09 |
| BRCA2 | Germline | Frame_Shift_Del | TCGA-13-1498-01A-01W-0549-09 |
| BRCA2 | Germline | Frame_Shift_Del | TCGA-13-1499-01A-01W-0549-09 |
| BRCA2 | Germline | Frame_Shift_Del | TCGA-20-1685-01A-01W-0633-09 |
| BRCA2 | Germline | Frame_Shift_Del | TCGA-23-1029-01B-01W-0639-09 |
| BRCA2 | Germline | Frame_Shift_Del | TCGA-23-1114-01B-01W-0633-09 |
| BRCA2 | Germline | Frame_Shift_Del | TCGA-29-1784-01A-02W-0633-09 |
| BRCA2 | Germline | Frame_Shift_Ins | TCGA-24-1463-01A-01W-0549-09 |
| BRCA2 | Germline | Frame_Shift_Ins | TCGA-13-2061-01A-01D-1526-09 |
| BRCA2 | Germline | In_Frame_Del | TCGA-29-1701-01A-01W-0633-09 |
| BRCA2 | Germline | Nonsense_Mutation | TCGA-04-1367-01A-01W-0492-08 |
| BRCA2 | Germline | Nonsense_Mutation | TCGA-24-2293-01A-01W-0799-08 |
| BRCA2 | Germline | Nonsense_Mutation | TCGA-13-1512-01A-01W-0545-08 |
| BRCA2 | Germline | Nonsense_Mutation | TCGA-13-0726-01A-01W-0372-09 |
| BRCA2 | Germline | Nonsense_Mutation | TCGA-24-1562-01A-01W-0553-09 |
| BRCA2 | Germline | Splice_Site | TCGA-24-1555-01A-01W-0552-10 |
| BRCA2 | Germline | Splice_Site | TCGA-25-1634-01A-01W-0615-10 |
| BRCA2 | Germline | Splice_Site | TCGA-13-0793-01A-01W-0370-10 |
| BRCA2 | Germline | Splice_Site | TCGA-24-0975-01B-02W-0486-08 |
| BRCA1 | Somatic | Nonsense_Mutation | TCGA-04-1357-01A-01W-0492-08 |
| BRCA1 | Somatic | Nonsense_Mutation | TCGA-13-0730-01A-01W-0371-08 |
| BRCA1 | Somatic | Splice_Site | TCGA-13-0761-01A-01W-0370-10 |
| BRCA1 | Somatic | Missense_Mutation | TCGA-13-0804-01A-01W-0372-09 |
| BRCA1 | Somatic | Frame_Shift_Ins | TCGA-13-1489-01A-01W-0549-09 |
| BRCA1 | Somatic | Frame_Shift_Del | TCGA-23-1026-01B-01W-0484-10 |
| BRCA1 | Somatic | Frame_Shift_Ins | TCGA-24-1846-01A-01W-0639-09 |
| BRCA1 | Somatic | Nonsense_Mutation | TCGA-24-1847-01A-01W-0633-09 |
| BRCA1 | Somatic | Frame_Shift_Del | TCGA-24-2035-01A-01W-0722-08 |
| BRCA1 | Somatic | Nonsense_Mutation | TCGA-25-1625-01A-01W-0615-10 |
| BRCA1 | Somatic | Frame_Shift_Del | TCGA-25-1630-01A-01W-0615-10 |
| BRCA1 | Somatic | Frame_Shift_Ins | TCGA-25-1632-01A-01W-0615-10 |
| BRCA1 | Somatic | Frame_Shift_Del | TCGA-29-1768-01A-01W-0633-09 |
| BRCA1 | Somatic | Frame_Shift_Del | TCGA-29-1776-01A-01W-0639-09 |
| BRCA1 | Somatic | Nonsense_Mutation | TCGA-29-2427-01A-01W-0799-08 |
| BRCA1 | Somatic | Nonsense_Mutation | TCGA-30-1857-01A-02W-0639-09 |
| BRCA1 | Somatic | Splice_Site | TCGA-36-2534-01A-01D-1526-09 |
| BRCA1 | Somatic | Missense_Mutation | TCGA-42-2591-01A-01D-1526-09 |
| BRCA1 | Somatic | Nonsense_Mutation | TCGA-61-1914-01A-01W-0639-09 |
| BRCA2 | Somatic | Nonsense_Mutation | TCGA-04-1331-01A-01W-0486-08 |
| BRCA2 | Somatic | Nonsense_Mutation | TCGA-09-2050-01A-01W-0799-08 |
| BRCA2 | Somatic | Missense_Mutation | TCGA-13-0792-01A-01W-0370-10 |
| BRCA2 | Somatic | Frame_Shift_Del | TCGA-13-0885-01A-02W-0421-09 |
| BRCA2 | Somatic | Frame_Shift_Del | TCGA-13-0890-01A-01W-0421-09 |
| BRCA2 | Somatic | Frame_Shift_Del | TCGA-13-1481-01A-01W-0549-09 |
| BRCA2 | Somatic | Missense_Mutation | TCGA-23-1030-01A-02W-0486-08 |
| BRCA2 | Somatic | Frame_Shift_Del | TCGA-23-1120-01A-02W-0484-10 |
| BRCA2 | Somatic | Missense_Mutation | TCGA-24-1103-01A-01W-0488-09 |
| BRCA2 | Somatic | Frame_Shift_Del | TCGA-24-1555-01A-01W-0552-10 |
| BRCA2 | Somatic | Frame_Shift_Del | TCGA-29-1693-01A-01W-0633-09 |
| BRCA2 | Somatic | Nonsense_Mutation | TCGA-29-1762-01A-01W-0633-09 |
| BRCA2 | Somatic | Missense_Mutation | TCGA-61-1903-01A-01W-0639-09 |
